# Comparative Evaluation of Relapse After LeFort 1 Osteotomy in Cleft Palate and Non-cleft Individuals: A Longitudinal Study

**DOI:** 10.7759/cureus.52298

**Published:** 2024-01-15

**Authors:** Shruti Patil, Shrinivas M Basavaraddi, Ameet V Revankar, Roopak D Naik, Anil Desai, Niranjan Kumar, Balaram Naik

**Affiliations:** 1 Department of Orthodontics and Dentofacial Orthopedics, Shri Dharmasthala Manjunatheshwara (SDM) College of Dental Sciences and Hospital, A Constituent Unit of Shri Dharmasthala Manjunatheshwara University, Dharwad, IND; 2 Department of Oral and Maxillofacial Surgery, Shri Dharmasthala Manjunatheshwara (SDM) College of Dental Sciences and Hospital, A Constituent Unit of Shri Dharmasthala Manjunatheshwara University, Dharwad, IND; 3 Department of Plastic Surgery, Shri Dharmasthala Manjunatheshwara (SDM) College of Medical Sciences and Hospital, A Constituent Unit of Shri Dharmasthala Manjunatheshwara University, Dharwad, IND; 4 Department of Conservative Dentistry and Endodontics, Shri Dharmasthala Manjunatheshwara (SDM) College of Dental sciences and Hospital, A Constituent Unit of Shri Dharmasthala Manjunatheshwara University, Dharwad, IND

**Keywords:** lefort 1 osteotomy, lateral cephalometry, cleft lip and palate, relapse, alveolus and palate

## Abstract

Purpose: The aim of this study was to assess and compare the likelihood of relapse one year after LeFort I advancement surgery in patients with and without cleft lip and palate.

Methods: A retrospective observational study which included two groups of participants who underwent LeFort I maxillary advancement was performed. Group 1 included 10 non-cleft subjects and Group 2 included 21 subjects with cleft palate. These maxillary deficient patients were chosen and operated using a technique where only a sagittal displacement was intended. Patients who underwent additional mandibular surgery, significant vertical or transverse alterations, or both were excluded. Pre-operative (T1), immediately post-operative (T2), and minimum one-year follow-up (T3) lateral cephalograms were studied for each group. Skeletal stability and dental stability after LeFort I surgery at a minimum of one-year follow-up in cleft palate and non-cleft patients were evaluated.

Results: For the given sample size, relapse tendencies showed statistically significant differences between cleft palate patients and non-cleft palate patients after maxillary advancement. The sella nasion angle and horizontal overlap of the maxillary and mandibular incisors (overjet) decreased by 2 degrees and 0.9 mm respectively in the cleft palate group while decreasing by 1.10 degrees and 0.40 mm in the non-cleft group.

Conclusions: After maxillary advancement with LeFort I osteotomy and miniplate fixation in patients with cleft palate and non-cleft patients, some degree of relapse was detected in both groups for the given sample size after one year post-operatively. The cleft palate group displayed additional relapse tendencies when compared to the non-cleft group.

## Introduction

Ross noted that individuals with cleft lip and palate typically exhibit skeletal class III malocclusions with varying degrees of maxillary hypoplasia [1]. The most popular surgical treatment used to treat maxillary skeletal hypoplasia is LeFort I maxillary advancement [2,3]. LeFort I osteotomy is a horizontal maxillary osteotomy utilized in the correction of midface deformities allowing movement anteriorly/posteriorly, vertically, rotationally, and with segmentation. The technique gained popularity throughout the 1970s as a result of its ability to handle discrepancies in all three planes of space [2,3]. Because of its adaptability and lack of adverse consequences, the LeFort I osteotomy is the method of choice for Class III patients with maxillary hypoplasia. The growing adoption of this strategy has also been influenced by aesthetic factors [4].

Individuals with cleft palate provide unique challenges in the surgical treatment of maxillary deficiency, and it is reasonable to anticipate whether they will differ from patients without clefts in terms of post-operative skeletal stability and soft tissue changes [5]. Relapse tendency, in which the operated jaw recedes partially or entirely to its original position after orthognathic surgery, is the worst side effect [6]. The likelihood of relapse may be influenced by a number of factors including the cleft itself, surgery, fixation method, neuromuscular adaptation, and orthodontics [5]. The distinction between a total or partial relapse and relapse tendency is hinted at in earlier literature, although this term has not yet been given a precise definition [6]. Clinically measuring the degree of relapse involves observing occlusal changes [6]. For patients with cleft palate in particular, skeletal and dental stability is essential for satisfying long-term outcomes because scarring would make the procedure more difficult by limiting how far the surgery could advance and increase the likelihood of relapse [7].

Some studies have claimed excellent stability [5,8] but others have reported significant post-operative changes [5,9]. Factors proposed as having an influence on stability include the type of fixation [5,9,10], amount of repositioning [5,11], and use of bone grafts [5,12,13].

The aim of this investigation was to evaluate and compare the amount of relapse in cleft palate and non-cleft palate patients after a minimum time period of one year post-operatively.

## Materials and methods

Sample selection

This retrospective observational study was approved by the Institutional Review Board of Shri Dharmasthala Manjunatheshwara College of Dental Sciences and Hospital, Dharwad, Karnataka, India.

Inclusion criteria

**Figure 1:**
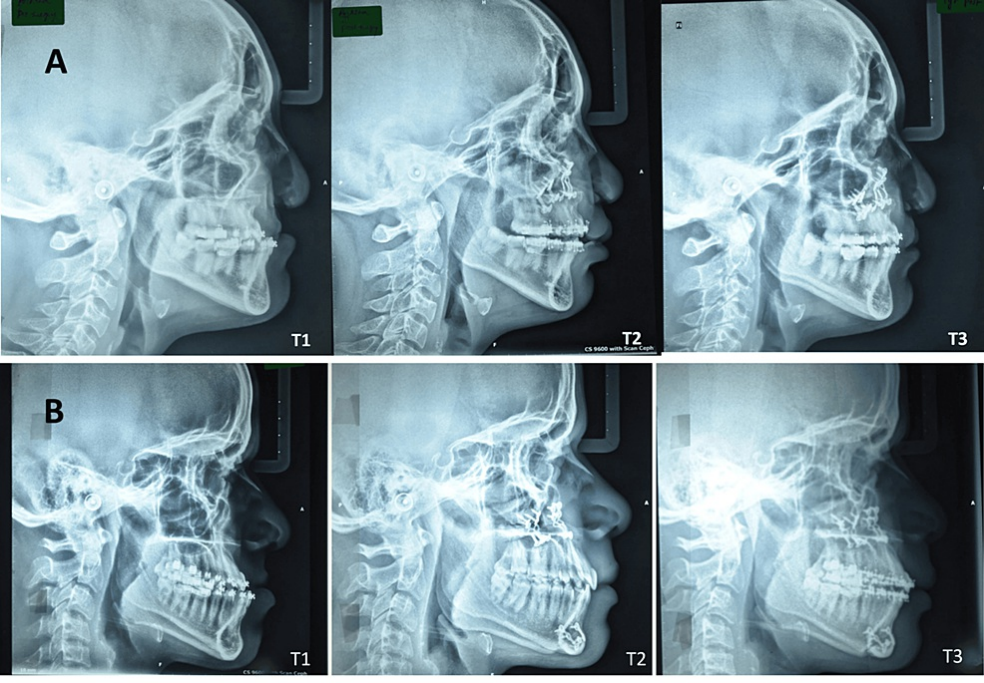
Lateral cephalograms of the three timepoints pre-operative (T1), immediate post-operative (T2), and one-year follow-up (T3) of (A) cleft lip and palate patients and (B) non-cleft patients

Patient records in the age range of 18 to 33 years submitted to orthodontic treatment and orthognathic surgery for maxillary advancement with the LeFort I maxillary down fracture technique operated at our craniofacial unit were included in this study after patient's consent. Lateral cephalograms (total of 93) at the following time points of patients with cleft lip and palate and non-cleft were taken pre-operative (T1), immediate post-operative (T2), and follow-up records (minimum one year) after surgery (T3) (Figure 1).

Exclusion criteria

Patients who had undergone bimaxillary surgery and patients having syndromes causing facial deformity that required maxillary advancement surgery were excluded.

In conformity to the objective patients who had undergone maxillary advancement alone were selected and those with bimaxillary surgeries were excluded as the bimaxillary surgery would act as a confounding factor in assessing the relapse because of two reasons: (a) In a bimaxillary surgery, the amount of maxillary advancement is limited as the jaw movements are split up between the maxilla and mandible; this could affect the amount of relapse in comparison to a maxillary advancement alone where the discrepancy would be corrected by maxillary advancement alone and (b) The assessment of relapse in bimaxillary surgery would require a different method of cephalometric assessment than the assessment in maxillary advancement alone and therefore combining the two in the same group would not be feasible.

Further patients who underwent significant vertical or transverse alterations during maxillary advancement surgery were not included since the relapse tendencies in the vertical and transverse dimension differ from that in the sagittal dimension [6] with greater relapses being reported with vertical and transverse dimension changes [6].

Records of 31 patients who underwent maxillary advancement surgery were collected, which included 10 non-cleft patients and 21 patients with cleft lip, alveolus, and palate. 

Due to the retrospective design of the study and only selecting cases where maxillary advancement surgery alone was performed, excluding cases in which maxillary advancement was performed along with mandibular surgery to limit the effect of confounding variables as explained above, the number of patients in the non-cleft group was lesser which caused unequal sample distribution between the two groups. In Tables 1, 2, demographic information is displayed.

**Table 1:** Demographic details of age and sex of the patients with cleft

Maxillary deficiency	Age (Years)			Sex	
	Min	Max	Average	Male	Female
With cleft	18	33	25.50	8	13

**Table 2:** Demographic details of age and sex of the patients without cleft

Maxillary deficiency	Age (Years)			Sex	
	Min	Max	Average	Male	Female
Without cleft	18	33	25.50	5	5

**Figure 2:**
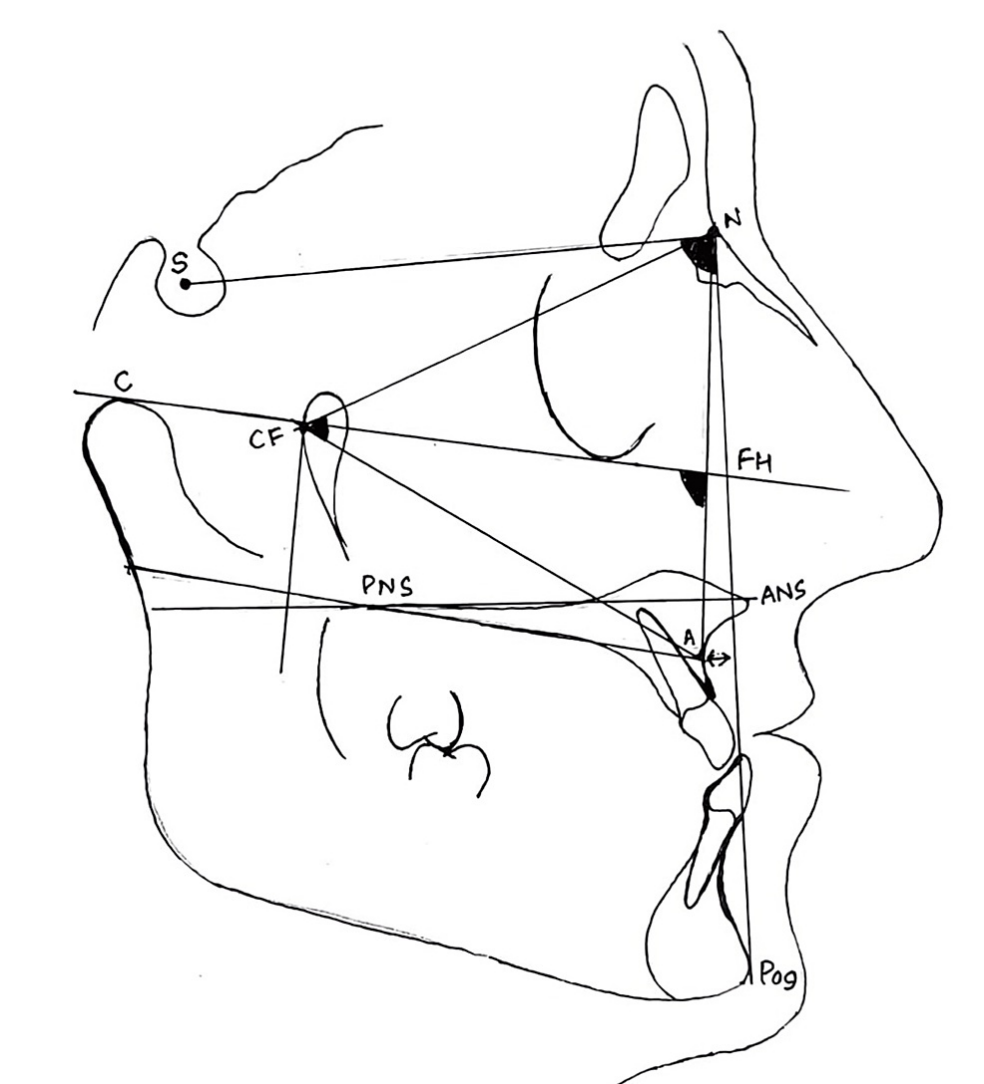
Cephalometric landmarks and parameters A straight line that connects the center of the point Sella (S), the point nasion (N), and the point A was used to calculate the maxillary anteroposterior location (SNA). The skeletal alterations were measured using parameters SNA, FH/N-A, A/N-Pog, N-CF-A and FH/ANS-PNS. The degree of the maxillary basal arch at its anterior limit relative to the total facial profile was measured by A-Npog. The angle of convexity (A-Npog) is formed by the intersection of the line N-point A to point A-pog. N-CF-A measures the maxillary height. CF point is formed by the intersection of the FH and the perpendicular through the Pt point. The maxillary height was measured by the angle formed from the planes CF-A and CF-N. The inclination of the maxilla to the cranium (FH-ANS-PNS) was measured by the angle formed by the palatal plane to the Frankfort horizontal plane which is formed by the line connecting porion to orbitale. Maxillary depth or the horizontal relationship of the maxilla to the cranium was measured by the angle formed by the planes N-A and FH.

Each patient underwent a typical LeFort I osteotomy in one piece. On the lateral surfaces of the maxilla, reference lines were drawn before the surgical cuts. Following mobilization, the maxilla was relocated to its intended location by following reference lines. The mandibular and maxillary arches were stabilized with surgical splints prior to maxillary fixation. On either side of the maxilla, two L-shaped mini plates were used for rigid intermaxillary fixation (RIF). The plating system employed 1 mm plates and four screws, two above and two below the surgical cut, each with a diameter of 2.0 mm. The occlusal splint was removed after RIF, and the occlusion was examined. There was no utilization of post-operative maxillomandibular fixation, and no patient received a bone graft. After surgery, light elastics were sometimes used to help with occlusal correction. All the cephalograms were taken using a Kodak 9000C 3D cephalostat (Carestream Health Inc., New York, USA) with the Frankfort horizontal plane parallel to the floor with teeth in occlusion and the lips in a relaxed position. The same cephalostat was used for all the radiographs. One examiner evaluated photographs, lateral cephalometric radiographic tracings, and clinical assessments of occlusion at T1 (one week prior to surgery), T2 (one week after surgery), and T3 (minimum one year after surgery) to study skeletal and dental relapse in the horizontal and vertical planes. Figure 2 shows all the skeletal parameters included in the study.

The cephalometric parameters listed in Figure 2 were manually traced and measured by one examiner (S.P) to ensure standardization and compared to each other from the pre-operative, post-operative, and one-year post-operative tracings. Overjet and overbite values were also assessed. 

Statistical analysis

Statistical analysis was done using IBM SPSS Statistics for Windows, Version 22 (Released 2013; IBM Corp., Armonk, New York, United States). A descriptive analysis of the data was presented as mean and standard deviation (SD). A paired "t"-test was used to compare the means among the time interval levels one week pre-surgical, one week post-surgical, and one-year post-surgery (mean 3.5 years) within the groups, and an unpaired "t"-test was used for intergroup comparison.

## Results

Patients without clefts

**Figure 3:**
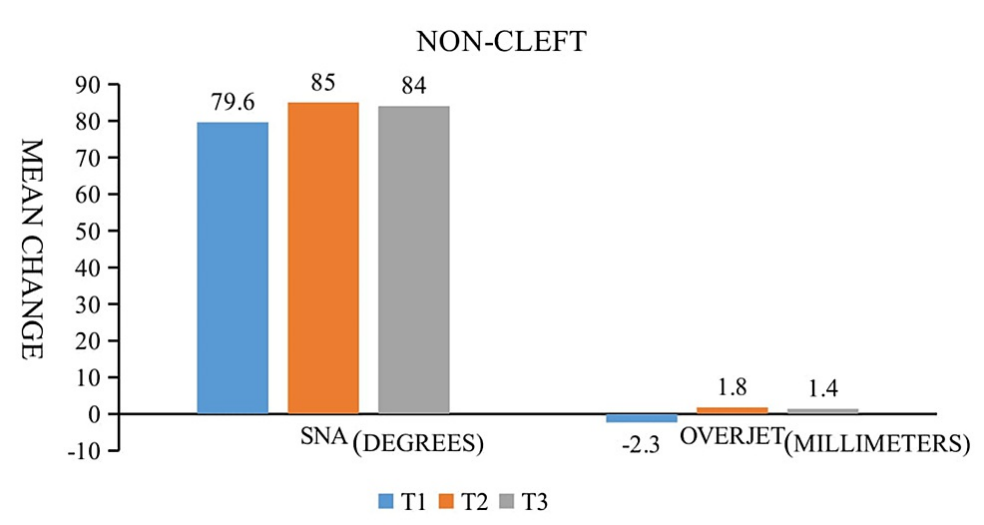
Maxillary deficiency in non-cleft patients. The bar graph depicting the mean change of SNA and overjet from T1 to T3

A total of 30 lateral cephalograms were assessed. For the group without clefts, skeletal and dental alterations from T1 to T2 are also shown in Table 3. A clear increase in all the parameters is noted from T1 to T2. SNA and maxillary depth significantly increased in this group from pre-operatively (T1) to post-operatively (T2) by 3.6 and 3.2 degrees, respectively. In addition, a statistically significant change in convexity was detected. The maxillary depth and sagittal improvement on average were 2.70 millimeters and 5.4 mm, respectively. The change in the maxillary height was 2.6 mm which was significant statistically (Table 4). The change in the palatal plane was 2.6 degrees. The average advancement in terms of overjet was 4.10 mm. A statistically significant amount of relapse was observed one year after surgery (Table 4). SNA and overjet both had declines of 1.10 degrees and 0.24 mm (Table 3 and Figure 3).

**Table 3:** Mean of parameters * statistically significant

Parameters	SNA (Degrees)	FH/NA (Degrees)	ANPOG (Degrees)	N-CF-A (Degrees)	FH-ANS (Degrees)	Overjet (mm)	Overbite (mm)
T1 Non-cleft	79.60	86.90	3.30	54.20	5.70	-2.30	1.55
T1 Cleft	72.38	80.62	-3.29	58.24	3.86	-3.17	1.83
T2 Non-cleft	85.00	89.60	-2.90	55.20	3.10	1.80	1.50
T2 Cleft	79.52	86.33	-3.29	56.90	4.14	2.55	1.69
T3 Non-cleft	83.9	88.30	-3.00	53.50	3.70	0.90	1.40
T3 Cleft	77.52	85.38	-3.62	56.48	4.48	1.64	1.45
T2-T1 Non-cleft	7.14	-2.70	6.20	-1.00	2.60	4.10	0.05
T2-T1 Cleft	5.4	5.71	9.48	1.33	0.29	5.71	0.14
T2-T3 Non-cleft	1.1*	1.3	0.1	1.7	-0.6	0.45*	0.1
T2-T3 Cleft	2*	0.95	0.33	0.43	-0.33	0.9*	0.24

Patients with clefts

**Figure 4:**
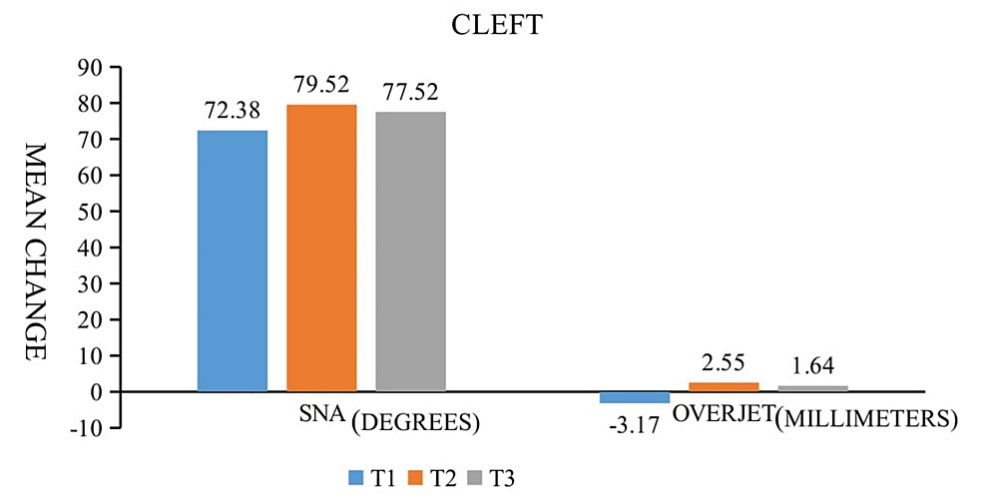
Maxillary deficiency in cleft patients.The bar graph depicting the mean change of SNA and overjet from T1 to T3

A total of 63 lateral cephalograms were assessed. Repeated evaluations of the intra-examiner reproducibility revealed no statistically significant difference. Table 3 demonstrates that all parameters used to measure sagittal movements have clearly increased from T1 to T2. SNA and maxillary depth increased from pre-operative (T1) to post-operative (T2) by 7.14 and 5.71 degrees, respectively. A significant change in the angle of convexity was noted. Overjet served as a gauge, showing an average advancement of 5.71 mm (Table 3 and Figure 4).

**Figure 5:**
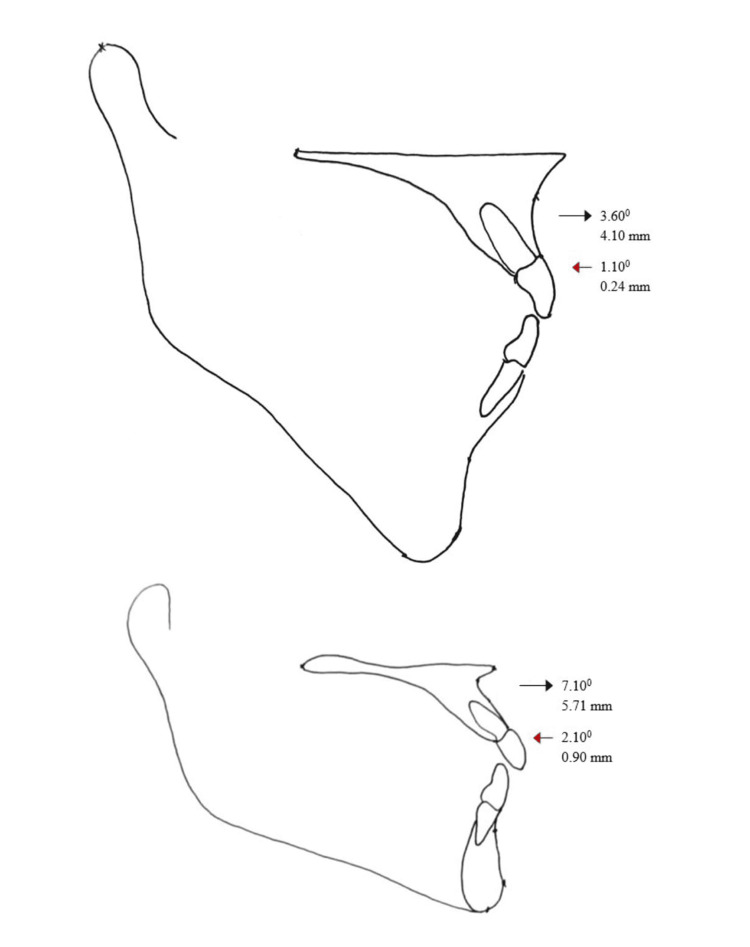
Mean changes in SNA (degrees) and Overjet (mm) and mean relapse at 3.5 years (mean) at selected landmarks among 10 non-cleft patients (top) and 21 cleft patients (bottom) treated with surgical advancement of maxilla. Arrow (black) indicates change as a result of surgery and arrow (red) indicates relapse during follow-up

Although only sagittal movements had been planned, a certain amount of rotation with respect to the occlusion could not be avoided. These changes (FH/ANS-PNS) were not significant statistically. There was no change in maxillary height. After one year post-operatively, a certain amount of relapse was noted. SNA decreased by 2.1 degrees which was significant statistically (Table 4). With respect to dental parameters, statistically significant changes were observed in overjet at T3 (minimum one year of post-surgery) (Table 4). On average, there was a decrease in overjet of 0.90 mm (Table 3, Figures 4, 5).

**Table 4:** Significance(p) of skeletal and dental values comparing T1, T2, and T3 in non-cleft and cleft groups * statistically significant

Parameters	SNA	FH/NA	ANPOG	N-CF-A	FH-ANS	Overjet	Overbite
Non-cleft T2-T1	0.000*	0.009*	0.000*	0.904	0.039	0.000*	0.840
Cleft T2-T1	0.000*	0.000*	0.000*	0.109	0.721	0.000*	0.794
Non-cleft T2-T3	0.003*	0.207	0.885	0.131	0.382	0.010*	0.678
Cleft T2-T3	0.000*	0.059	0.628	0.480	0.645	0.035*	0.086

Comparison of the groups

To assess the effects of the sample size inequality on the test statistics, Levene's test for equality of variances was used. For one variable that reported a significant p-value on the Levene's test (FH-ANS), further inferential statistics were processed based on ‘equal variances not assumed “and the corresponding p-value for “equal variances not assumed" was considered (Table 5). The parameters for sagittal displacement namely SNA showed statistically significant differences at T3 (one-year post-surgery) with the measured values in the group with clefts being smaller (Table 5) indicating greater sagittal relapse in this group.

**Table 5:** Comparison of parameters between cleft and non-cleft patients T3 (one-year post-surgery) using the unpaired "t"-test $ “Equal Variances not assumed” as per results of Levene's test for equality of variances *Statistically significant

Parameters	Cleft	Non-cleft	Significance
SNA	77.52	83.90	0.0000*
FH/NA	85.38	88.3	0.066
ANPOG	-3.62	-3	0.0958
N-CF-A	56.48	53.5	0.0922
FH-ANS	4.48	3.7	0.4921^$^
Overjet	2.55	0.9	0.2264
Overbite	1.69	1.4	0.8669

## Discussion

At skeletal maturity, cleft maxillary hypoplasia is a frequent feature in patients with cleft palate. Between 11% and 60% of patients require LeFort I advancement surgery to treat cleft maxillary hypoplasia [1,14-17]. The severity of the cleft deformity at birth [18,19] palatal scar tissue from prior surgical repair [20-23], inherent genetic growth potential [24,25], missing bone, teeth, and soft tissue, the abnormal position of the mandible may also make the maxillary-mandibular skeletal discrepancy worse.

Maxillary advancement in patients with non-clefts

Relapse tendencies following maxillary advancement in non-cleft children are less common than in cleft patients, although they are nonetheless seen in a manner similar to previous studies. Both Teuscher and Sailer and Iannetti et al. found no evidence of post-surgical relapse [8,26]. In our studies, SNA and overjet reduced by 1.10 degrees and 0.40 mm, respectively (Table 3).

Although there was a statistically significant increase in parameters like SNA, maxillary height, maxillary depth, overjet, and angle of convexity, we also found a slight relapse tendency at T3 (minimum one-year post-surgery) (Table 4).

Maxillary advancement in patients with clefts

The rates of post-surgical relapse after orthognathic surgery in cleft lip and palate populations have been extensively investigated over the years [27]. Bony gaps may develop after excessive maxillary advancement, which could exacerbate relapse tendencies. Araujo et al. reported relapse rates of 0 to 5% in the group with bone grafting in the pterygomaxillary gap [9]. The study by Eggert et al. produced superior results after miniplate fixation, in cleft patients [28]. Relapse rates in patients with unilateral cleft lip and palate range from 19% to 32% for horizontal movements and from 19% to 52% for vertical movements [7]. Relapse rates after orthognathic surgery are much greater in cleft populations than in non-cleft populations, and various contributing factors, including palatal scarring, poor vascularity, soft tissue tension, the existence of fistulas, and inadequate dentition, have been linked to these higher rates [6]. In our study after maxillary advancement by Lefort I osteotomy and miniplate fixation in patients with complete clefts of the lip, alveolus, and palate, SNA decreased by 2 degrees and overjet decreased by 0.9mm on average minimum 1 year after surgery (mean 3.5 years). Heliovaara et al. have found a horizontal relapse rate of 0.8 mm similar to our study [29]. Velasques et al. found a horizontal relapse rate of 1.2 mm similar to our study [30]. In contrast to other studies, we could not find statistically significant relapses in the vertical dimension (Table 4) [6].

Comparison of both the groups

Relapse tendencies rely on a variety of factors, and many investigations have shown conflicting results, particularly in cases of cleft palate. There are few studies carried out for the Indian population to determine whether there is a difference in a relapse in cleft cases compared to non-cleft patients, despite the fact that miniplate fixation has grown more popular in LeFort I osteotomy. The percentage of non-acceptable outcomes in clefts was 48%, compared to 20% in non-clefts, according to Freihofer, although Nissen and Schmidseder found no significant differences between the two groups [31]. Relapse tendencies are widely recognized to begin as early as six months have elapsed from surgery [6]. According to Araujo et al., Epker, Teuscher and Sailer, Persson et al., and Houston et al. circumstances can be deemed stable after around a year post-surgery [8,9,12,14,32]. According to Ayliffe et al. [33], 86% of subjects with cleft lip and palate had vertical measures that relapsed from 0 to 2.5 mm, whereas Kumari et al. studied subjects without cleft lip and palate and observed horizontal and vertical relapses of 21.63 and 41.54%, respectively [34].

The present study aims to examine and compare the relapse tendencies after LeFort I advancement a minimum of one-year after surgery in cleft and non-cleft patients. Skeletal changes pre-operative (T1) after maxillary advancement (T2) and minimum period of one year post-operative (T3) were assessed using parameters SNA, FH/N-A, A/N-Pog, N-CF-A, and FH/ANS-PNS (Figure 1). A clear increase of all parameters measuring sagittal movements was noticed from preoperatively (T1) to post-operatively (T2), respectively, which is significant (Table 4). The mean sagittal improvement in the cleft and the non-cleft group was 7.14 and 5.4 degrees, respectively with an average advancement of 5.71 mm and 4.10 mm respectively (Table 3, Figures 3, 4).

After a minimum time period of one year post-operatively, a certain amount of relapse was noted cephalometrically. A sagittal relapse of 2 degrees and 1 degree was seen in the cleft and non-cleft groups, respectively (Figure 5).

In the intergroup comparison, the parameter for sagittal displacement SNA showed statistically significant differences at T3 (one-year post-surgery) with the measured values in the group with clefts being smaller (Table 5) indicating greater sagittal relapse in this group.

The larger amount of advancement to attain a normal dimension might be a contributing factor to the larger relapse tendency in the cleft group [6]. Scarring from preceding surgery can reduce mobilization of the maxilla during osteotomy and influence postoperative stability.

Limitations of the study 

This investigation is limited in its power by the sample size number and inhomogeneous group distribution (cleft palate 21 vs. non-cleft palate 10). The lesser number of patients in the non-cleft group was because only cases where maxillary advancement surgery alone was performed were taken into account and those cases in which maxillary advancement was performed along with mandibular surgery were excluded to limit the effect of confounding variables as explained in the inclusion/exclusion criteria. 

Since the design of the study is retrospective observational, the existence of confounding variables and their effect on the results cannot be denied. The following confounders may have an effect on the generalizability of the results obtained in this study: (a) The amount of maxillary advancement performed in each of these patients varied from a minimum of 4mm to a maximum of 8mm (mean 6mm) for 22 patients. For nine patients, the data on the quantum of maxillary advancement were not available. This could have an effect on the results as there is a positive correlation between the amount of maxillary advancement and horizontal relapse [35], (b) The follow-up records at T3 were obtained at variable time from the surgery minimum being one year and maximum being 10 years (mean 3.5 years). This wide variation occurred because the post-treatment follow-up records are not obtained routinely as an institutional policy and therefore were not available retrospectively rather, they were obtained by recalling patients in the database at the time of the study. The minimum period of 1 year was selected since the maximum amount of relapse following maxillary advancement surgery occurs between 6 months and 1 year [15]. Therefore, the minimum period of 1 year selected mitigated the effect of this variable time period follow-up, and (c) The type of maxillary advancement surgery could be a potential confounder however this was taken into account, and only patients whose sagittal displacement was included. Patients who underwent significant vertical or transverse alterations or both were not included. Therefore, the effect of this confounder was eliminated. 

Additional surgery in the mandible and patients who underwent significant alterations in the vertical and transverse dimensions during maxillary advancement surgery could act as potential confounding factors as described previously; however, these patients who underwent additional mandibular procedures/significant transverse/vertical dimension changes during maxillary advancement surgery were excluded. Therefore, the effect of these confounders was eliminated.

## Conclusions

After maxillary advancement with LeFort I osteotomy and miniplate fixation in patients with cleft palate and non-cleft patients, some degree of relapse was seen in both the groups for the given sample size after a minimum period of 1 year post-operatively. The cleft palate group displayed additional relapse tendencies when compared to the non-cleft group. This should be taken into consideration when planning maxillary advancement surgery on these patients warranting overcorrection, in patients with clefts commensurate with the amount of relapse expected.
